# Overexpression of Interleukin-17 Modulates Responses to Marek’s Disease Virus Infection and Tumor Formation in Chickens

**DOI:** 10.3390/v17071009

**Published:** 2025-07-18

**Authors:** Nitish Boodhoo, Katherine Blake, Fatemeh Fazel, Janan Shoja Doost, Shayan Sharif

**Affiliations:** Department of Pathobiology, Ontario Veterinary College, University of Guelph, Guelph, ON N1G 2W1, Canada; boodhoon@uoguelph.ca (N.B.); kblake@uoguelph.ca (K.B.); ffazel@uoguelph.ca (F.F.); jshojado@uoguelph.ca (J.S.D.)

**Keywords:** MDV, chickens, Th17 cells, IL-17A, adaptive immunity

## Abstract

Marek’s Disease Virus (MDV) is a highly contagious pathogen in chickens, resulting in immunosuppression and T-cell lymphomas. Understanding the role of host cytokines in MDV pathogenesis is crucial for developing effective interventions. This study investigated the in vivo effects of overexpressing avian interleukin-17 (IL-17) in Marek’s disease virus infection model and its impact on T-cell populations. We utilized a recombinant pCDNA3.1 plasmid that expresses IL-17 at days 4 and 10 post-MDV infection in chickens. Our findings demonstrate that IL-17 overexpression significantly enhanced MDV replication. However, treatment with the plasmid expressing IL-17 led to a reduction in MD disease severity. Additionally, IL-17 treatment markedly altered the frequency of CD4+ and CD8α+ αβ T-cells. Specifically, at 21-dpi, there was an increase in CD3+ CD8α+ αβ T cells and a decrease in CD3+ CD4+ αβ T-cells within the spleen of chickens treated with the plasmid expressing IL-17. These modulatory effects suggest a possible mechanism by which IL-17 facilitates immune system cell activation and enhances viral persistence. This study underscores the pivotal role of IL-17 in MDV infection dynamics and offers.

## 1. Introduction

Among the many factors that form part of an antiviral response, interleukin-17 (IL-17) is implicated in diverse immune responses and the pathogenesis of several diseases [1,2,3]. IL-17 signaling through expression of the IL-17 receptor (IL-17R) on various epithelial cells and immune system cells can lead to diverse outcomes [4]. IL-17 can modulate differentiation of the distinct T-helper (Th)1 and Th17 T-cell lineages. At specific sites of infection or inflammation, IL-17 can enhance the recruitment of T cells by inducing the expression of chemokines and adhesion molecules [2]. Additionally, IL-17 may influence the function of interferon gamma (IFN-γ) producing CD8 T-cells, impacting their cytotoxic potential [5,6].

The interplay between IL-17 and IFN-γ has been documented across a spectrum of pathological mechanisms, including those involved in infectious diseases, cancer, and autoimmune disorders. In our previous studies using a Marek’s disease virus (MDV) infection model, we observed a decrease in the percentage of IFN-γ+ T-cells and an increase in IL-17+ T-cells within the spleen [7,8,9]. Administration of either a recombinant chicken IL-17A or IFN-γ prior to infection with MDV and in combination with a vaccine has led to a reduction in Marek’s disease (MD) severity [8,10]. MDV, the causative agent of MD, is an *alphaherpesvirus* that targets T cells [11]. MDV pathogenesis has four phases in susceptible birds, outlined as follows: First, there is an early cytolytic phase within 2–7 days post-infection (dpi), which manifests as a semi-productive lytic viral replication in lymphocytes [12]. This is followed by a latency phase between 1– and 2-weeks post-infection in the CD4+ αβ T-cell subset that results in systemic viral dissemination [13]. MDV reactivation in CD4+ αβ T-cells initiates a late cytolytic and immunosuppressive phase from 2- to 3-weeks post-infection. Finally, a proliferative phase around 3 weeks is characterized by the formation of tumors that originate from CD4+ T-cell lymphomas [14]. The aberrant production of inflammatory and regulatory cytokines in MD can promote viral replication, immunosuppression, neurological disorders, and a lymphomatous proliferative disease that originates from CD4+ T-cell transformation [11,12,15]. Given the complex life cycle of MDV in chickens, modulating the host’s immune response before or during the early stages of infection may help directly eliminate infected or abnormal self-cells [16]. While both CD4+ and CD8+ T cells have been identified and implicated in MDV infection and disease progression, the importance of CD8+ T cells in antiviral immunity, tumor surveillance, and immune homeostasis is well-established [17,18]. The CD8 T-cell-mediated immune response is considered pivotal in controlling Marek’s disease [12]. Therefore, identifying strategies that focus on improving CD8+ T-cell function is highly sought after.

Recent studies in mammalian systems have revealed that IL-17 not only enhances CD8+ T-cell cytotoxic function but also contributes to antiviral immunity by modulating the immune environment at infection sites [19]. While the presence of IL-17+ T cells and their receptors has been demonstrated in chickens, particularly within intestinal intraepithelial lymphocytes (IELs), the functional role of IL-17 in avian immune responses remains largely unexplored. This gap is particularly relevant in the context of MD, where cytokine dysregulation is a key driver of viral persistence, immunosuppression, and tumor formation. Given that CD8+ T cells play a central role in antiviral immunity and tumor surveillance during MDV infection, understanding the interplay between IL-17 and CD8+ T cells could reveal novel mechanisms of immune modulation in chickens. Therefore, this study investigates the impact of IL-17 on cytokine expression and tumor lesion severity at distinct stages of MDV pathogenesis. By utilizing a plasmid overexpressing recombinant IL-17 in MDV-infected chickens, we aim to uncover potential therapeutic strategies for enhancing antiviral and antitumor immunity in poultry.

## 2. Materials and Methods

### 2.1. Plasmid Preparation

Cloning and functional activity of the chicken IL-17 has been previously demonstrated [8]. In brief, DH5α-competent bacterial cells were transformed with the pCDNA3.1/V5-HIS TOPO and pCDNA3.1/rchIL-17A-V5-HIS TOPO plasmids to propagate the respective plasmids for purification with a midi-prep kit (Qiagen, Toronto, ON, Canada). The recombinant plasmid overexpressing chicken IL-17 was named prchIL-17. After propagation and extraction, prchIL-17 was diluted in PBS and stored on ice prior to inoculation in chickens.

### 2.2. Virus Preparation

The very virulent MDV-RB1B strain (vvMDV) was provided courtesy of Dr. K.A. Schat (Cornell University, Ithaca, NY, USA) [20]. vvMDV-RB1B virus titers were calculated using primary chicken kidney cells obtained from 2- to 3-week-old specific pathogen-free (SPF) chickens to establish infectious doses of inoculum, as well as stocks (liquid nitrogen storage) [21].

### 2.3. Experimental Design and Sampling

*(i) Experimental Animals:* One-day-old SPF chickens (*n* = 280) were purchased from the Animal Diseases Research Institute, Canadian Food Inspection Agency (Ottawa, ON, Canada), and were accommodated within Horsfall units in the isolation unit at the University of Guelph. For the duration of the experiments, all chickens were given ad libitum access to food and water. All experiments were approved by the Animal Care Committee of the University of Guelph and were conducted according to their guidelines.

**Figure 1:**
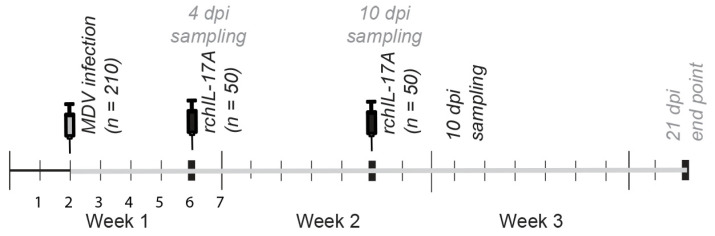
Schematic representation of the experimental timeline: Two-day-old chicks were infected via the intra-abdominal route with 250 PFU of a vvRB1B-MDV. At either 4- or 10dpi, different groups of chicks were inoculated intramuscularly via the thigh with 10 μg of either the pCDNA3.1/V5-HIS-TOPO or pCDNA3.1/V5-HIS-TOPO-rchIL-17A plasmid (prchIL-17) per chick. Chicks were euthanized at 10- and 21-dpi and whole spleen, lung, and skin samples were collected for FACS and real-time PCR analysis. At 21-dpi, tumor lesions were assessed in all infected groups.

*(ii) Experimental Outline:* Two-day-old chicks (*n* = 108) were infected with 250 plaque-forming units of the vvMDV-RB1B strain or received the sham/diluent treatment via the intra-abdominal route [10]. On days 4- and 10-post-infection, two groups of chicks were inoculated intramuscularly with either 10 μg/chick of the pCDNA3.1/V5-HIS-TOPO empty vector (*n* = 72) or prchIL-17 (*n* = 72). The intramuscular inoculum of 10 μg of plasmid/chick was previously optimized [22]. In this trial, the optimal plasmid dose (10 μg) and administration days (4- and 10-dpi) are illustrated (Figure 1).

*(iii) Sampling:* At 4-, 10-, and 21-days post-infection (dpi), tissue samples were collected for various analytical processes. Chicks (*n* = 10/group) were euthanized at 4-, 10-, and 21-dpi and whole spleens were collected in PBS containing penicillin (10 U/mL) and streptomycin (10 μg/mL). Spleen samples were either stored in RNA*later* (*n* = 5/group) for RNA extraction or stored on ice (*n* = 5/group) for mononuclear cell isolation.

### 2.4. Spleen Mononuclear Cell Preparation and Stimulation

Whole spleens (*n* = 5/group) collected aseptically in PBS at various time points were washed twice before being crushed through 40-μm BD cell strainers (BD Biosciences, Mississauga, ON, Canada) using the rubber end of a 10-mL syringe plunger and subsequently washed in RPMI 1640 with penicillin (10 U/mL) and streptomycin (10 μg/mL) [23]. A gradient suspension was prepared by layering the mixture (2:1) onto Histopaque 1077 (Millipore-Sigma, Oakville, ON, Canada) and centrifuged at 600× *g* with no brakes for 20 min to allow for the separation of mononuclear cells. Buffy coats were aspirated and washed (2×) at 400× *g* for 5 min in RPMI 1640 with penicillin (10 U/mL) and streptomycin (10 μg/mL). Mononuclear cells were subsequently suspended in complete RPMI cell culture medium containing 10% fetal bovine serum (Millipore-Sigma, Canada), penicillin (10 U/mL), and streptomycin (10 μg/mL). Viability and total cell numbers were calculated using a hemocytometer and the trypan blue exclusion method. Mononuclear cells were suspended in complete RPMI cell culture medium at a density of 5 × 10^6^ cells/mL and stored on ice.

### 2.5. RNA Extraction and Reverse Transcription

RNA was extracted from spleens using TRIzol following the manufacturer’s instructions and treated with DNA-free DNase (ThermoFisher Scientific, Mississauga, ON, Canada) as previously described [24]. The quality as well as the quantity of RNA was estimated using NanoDrop ND-1000 spectrophotometry (NanoDrop Technologies, Wilmington, DE, USA). Subsequently, 1 μg of purified RNA was reverse transcribed to cDNA using Oligo (dT) 12–18 primers (SuperScript II First-Strand Synthesis System; Invitrogen Life Technologies, Carlsbad, Ottawa, ON, Canada) according to the manufacturer’s recommended protocol. The resulting cDNA was diluted 1:9 in diethyl pyrocarbonate-treated (DEPC) water.

### 2.6. Real-Time Polymerase Chain Reaction (RT-PCR)

Quantitative real-time PCR with SYBR green was performed on diluted cDNA using a LightCycler 480 II (Roche Diagnostics GmbH, Mannheim, GER) according to the manufacturer’s recommendation. In brief, quantitative real-time PCR was conducted under the following conditions: initial denaturation was performed at 95 °C for 5 min, followed by 40 cycles of denaturation at 95 °C for 10 s (primer annealing temperatures are listed in Table 1), and extension at 72 °C for 20 s, with an end-point melt-curve analysis. The relative fold change of target genes was calculated by the 2−ΔΔCT method. The Ct value for each sample was normalized against the β-actin housekeeping gene for respective samples. The data represent the mean from five biological replicates, using previously defined primers [9].

### 2.7. Flow Cytometry

Following a wash and a spin of 400× *g* for 5 min in FACS staining buffer (PBS with 0.5% Bovine Serum Albumin; BSA), 5.0 × 10^5^ spleen mononuclear cells were counter-stained for 15 min at 4 °C with anti-chicken specific antibodies. The antibodies used for the T-cell panel (mouse anti-chicken CD3ε-PB, mouse anti-chicken CD4-PE-CY7, mouse anti-chicken CD8α-FITC, and mouse anti-chicken γδTCR-PE were all purchased from Southern Biotech, Birmingham, AL, USA) are listed. Following a final wash and a spin of 400× *g* for 5 min, mononuclear cells were counterstained with non-fixable live/dead marker 7-AAD (BDTM Pharmingen, Mississauga, ON, Canada) and acquired for analysis using a BD FACS Canto II. All samples were acquired on the same day, and data were processed by FlowJo software, version 10.

### 2.8. Statistical Analysis

Graph-Pad Prism software, version 8 for Windows, was utilized to generate graphs and perform statistical analysis. All data were analyzed by the Kruskal–Wallis non-parametric test, Wilcoxon’s test (Mann–Whitney), or Fisher’s exact test, with the results presented as the mean ± standard deviation (SD). The results were considered statistically significant at *p* < 0.05 (*).

## 3. Results

### 3.1. IL-17 Treatment at 4-Dpi Decreases Disease Severity but Not Tumor Incidence

**Figure 2:**
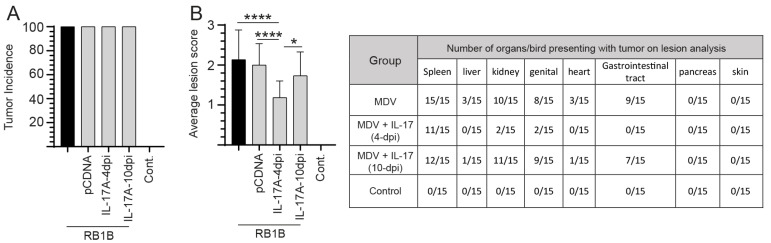
MDV tumor incidence at 21-dpi following treatment at 4- and 10-dpi with prchIL-17. Assessment of MD disease severity in vvMDV-RB1B-infected chickens based on presence of gross tumor lesions. (**A**) Tumor incidence in the various groups was calculated by observing gross tumors in visceral organs of birds (*n* = 105) at 21-dpi. (**B**) Assessment of lesion scores in different treatment groups based on the number of visceral and thoracic organs showing MDV-tumor lesions counted in each bird, with the average scores calculated. The table describes the number of chickens (*n* = 15/group) within each group presenting with tumor lesions. Fisher’s exact test or non-parametric Wilcoxon’s test (Mann-Whitney) was used to test significance, with the results shown as mean ± standard deviation. * (*p* ≤ 0.05) and **** (*p* < 0.001) indicates significant difference.

To demonstrate the effects of IL-17 administration in Marek’s disease, chicks were necropsied at 21-dpi to assess the presence of MD lymphomas (Figure 2). Tumor incidence defines the number of individual chickens that presented with tumors in at least one organ. One hundred percent of all chickens (15/15 chickens) infected with the vvMDV-RB1B virus developed MD tumors. No tumors were observed in the non-infected/control group (Figure 2A). The results demonstrate that the incidence of MD tumors was not reduced in chickens treated at either 4- or 10-dpi with prchIL-17 (Figure 2A).

While all infected groups had 100% tumor incidence, disease severity was affected following rchIL-17 treatment. Lesion scores represent the disease severity based on the presence of MD lymphomas in various organs. Most chickens assessed within the group treated with a plasmid expressing the rchIL-17 cytokine (prchil-17) at 4-dpi (11/15 chicks) but not at 10-dpi (0/15) did not present with visible tumors in the spleen at the end point analysis (21-dpi) (Figure 2B). Lesion scores were significantly lower in chickens treated with prchIL-17 at 4-dpi when compared to MDV-infected chickens (*p* ≤ 0.001), the empty vector control (*p* ≤ 0.001), or chickens treated with prchIL-17 at 10-dpi (*p* ≤ 0.05) that were subsequently infected with MDV (Figure 2B). Tumors were observed mainly in the spleen, kidney, and reproductive organs but not in the gastrointestinal tract. In comparison, the positive control group (MDV-infected) and the group treated with prchIL-17 at 10-dpi and then infected with MDV had visible tumors in various organs (Figure 2B). No differences in the lesion scores were observed between the positive control group and the group treated with the empty vector control, or between the positive control group and the group treated with prchIL-17 at 10-dpi and subsequently infected with MDV (Figure 2B).

To demonstrate how disease severity was affected following treatment with prchIL-17, a combination of FACS and real-time PCR was utilized to establish and describe changes in immune system cells and transcript levels of the MDV-*Meq* at 10- and 21-days post-infection.

### 3.2. IL-17 Treatment Increases RB1B Meq Expression in the Spleen, Lung, and Skin Tissues

**Figure 3:**
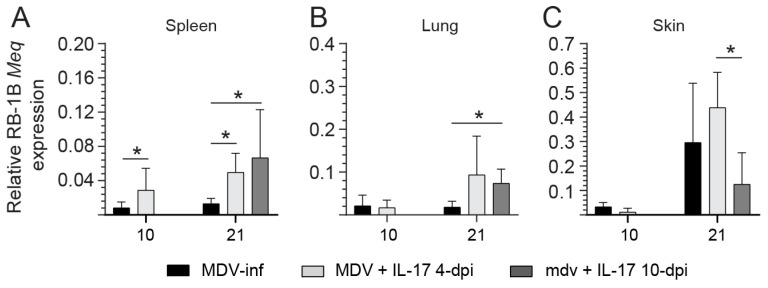
**vvMDV-RB1B viral role at 10- and 21-dpi.** vvMDV-*Meq* gene expression in the (**A**) spleen, (**B**) lung, and (**C**) skin tissues at 10- and 21-dpi as quantified by real-time-PCR and presented relative to β-actin expression. Non-parametric Wilcoxon’s test (Mann–Whitney) was used to test significance, with the results shown as mean ± standard deviation. * (*p* ≤ 0.05) indicates a significant difference.

We assessed the relative expression of RB1B *Meq* in the spleen, lung, and skin tissues of MDV-infected chickens at end point to determine the effect of rchIL-17 treatment. In the spleen, at 10-dpi, there was a significant increase (*p* < 0.05) in RB1B *Meq* expression when comparing MDV + prchIL-17 at 4-dpi to the MDV-infected group alone (Figure 3A). By 21-dpi, the expression levels were further elevated, with both rchIL-17 treated groups (MDV + prchIL-17 at 4-dpi and MDV + prchIL-17 at 10-dpi) showing significantly higher (*p* < 0.05) expression than the MDV-infected group.

In the lung tissue, the MDV + prchIL-17 10-dpi group had a significantly higher expression (*p* < 0.05) of RB1B *Meq* compared to the MDV-infected group at 21-dpi (Figure 3B). No significant differences in RB1B *Meq* expression were observed among the groups (MDV + rchIL-17 4-dpi or MDV + rchIL-17 10-dpi to the MDV-infected group alone) at 10-dpi (Figure 3B).

In comparison to 10-dpi, expression levels of RB1B *Meq* in skin tissue increased substantially by 21-dpi (Figure 3C). The MDV + prchIL-17 4-dpi group had a significant increase (*p* < 0.05) in RB1B *Meq* expression compared to the MDV + prchIL-17 10-dpi group. No significant differences in RB1B *Meq* expression were observed among the groups (MDV + prchIL-17 4-dpi to the MDV-infected group alone) at 10-dpi (Figure 3C).

### 3.3. IL-17 Treatment at 4-Dpi Increases the Frequency of CD8 T-Cells in the Spleen

**Figure 4:**
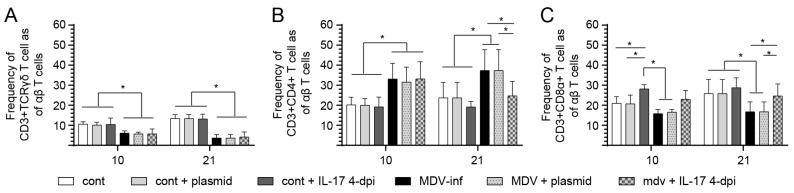
**T-cell frequency in vvMDV-RB1B-infected chickens treated at 4- and 10-dpi with prchIL-17.** FACS analysis to assess the frequency of the various T-cell subsets upon MDV infection post-treatment with the empty vector control plasmid or prchIL-17. At 10- and 21-dpi, the frequency of spleen (**A**) CD3 + CD8α+ γδ T-cells, (**B**) CD3+ CD4+, and (**C**) CD3+ CD8α+ αβ T cells are shown post-treatment with prchIL-17 and compared to non-infected chickens (*n* = 60) at 10- and 21- dpi. Non-parametric Wilcoxon’s test (Mann–Whitney) was used to test significance. Data represent mean ± SD (*n* = 5) at each time point. * (*p* < 0.05) indicates a significant difference. Cont.: control; MDV-inf: MDV-infected.

We assessed the frequency of γδ and αβ T-cell subsets within the spleen of MDV-infected chickens at 10- and 21-dpi following treatment with prchIL-17 at 4-dpi. Within CD3+ TCRγδ T-cells, there was a significant decrease (*p* < 0.05) in the frequency of CD3+ TCRγδ T-cells in all MDV-infected groups compared to the control groups at 10- and 21-dpi (Figure 4A). No differences in the frequency of CD3+ TCRγδ T cells were observed between the control and MDV-infected groups that were untreated or treated with prchIL-17 (Figure 4A).

Within αβ T-cells, treatment with prchIL-17 modulated the frequency of αβ T-cell subsets in both MDV- and non-infected chickens. At 10-dpi, the frequency of CD3+CD4+ αβ T-cells significantly increased (*p* < 0.05) in the MDV-infected group compared to the control group (Figure 4B). There was significantly (*p* < 0.05) more CD3+CD4+ αβ T-cells in the MDV-infected group compared to both the untreated and the control–plasmid groups (Figure 4B). At 21-dpi, there was a significant decrease (*p* < 0.05) in the frequency of CD3+ CD4+ αβ T cells in MDV- or non-infected groups treated with prchIL-17 compared to the control group (Figure 4C). At 10-dpi, the frequency of CD3+CD8α+ αβ T-cells was significantly higher (*p* < 0.05) in the MDV-infected group compared to the control group (Figure 4C). At 21-dpi, there were significant increases (*p* < 0.05) in the frequency of CD3+CD8α+ αβ T-cells in both the MDV-infected and the plasmid-pretreated MDV-infected groups compared to the control group (Figure 4C).

### 3.4. Treatment with IL-17 at 10-dpi Increases the Frequency of CD8+ T Cells in the Spleen of MDV-Infected Chickens

**Figure 5:**
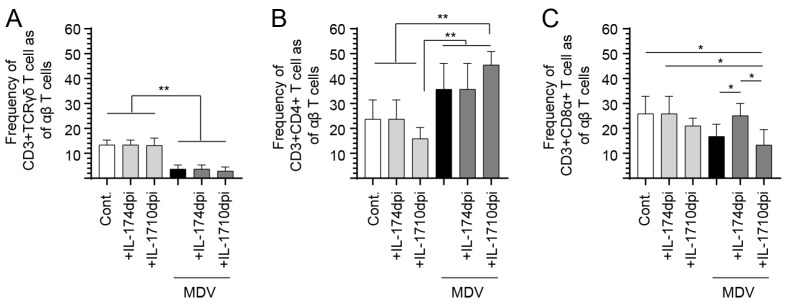
End point comparison for T-cell frequency between groups of chickens infected with the vvMDV-RB1B and treated at 4- and 10-dpi with prchIL-17. FACS analysis demonstrating a comparison of the frequency of the various T-cell subsets post-treatment with prchIL-17 in vvMDV-RB1B-infected chickens. End point splenocyte analysis showing the frequency of (**A**) CD3ε+CD8α+ γδ T-cells, (**B**) CD3ε+CD4+, and (**C**) CD3ε+ CD8α+ αβ T-cells only in MDV-infected chickens treated at 4- and 10-dpi with IL-17 (*n* = 60). Non-parametric Wilcoxon’s test (Mann–Whitney) was used to test significance. Data represent mean ± SD (*n* = 5) at each time point. * (*p* < 0.05) and ** (*p* < 0.01) indicate significant differences. Cont.: control; MDV-inf: MDV-infected.

**Figure 6:**
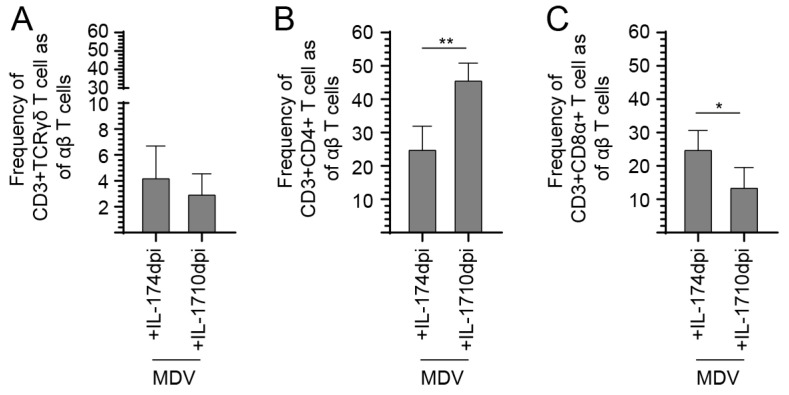
**Comparison of T-cell frequency in MDV-infected chickens treated with prchIL-17 at 4- and 10-dpi.** Comparison of the frequency of (**A**) CD3ε+ CD8α+ γδ T-cells, (**B**) CD3ε+CD4+ T-cells, and (**C**) CD3ε+CD8α+ αβ T-cells between groups of chickens that were treated at 4- and 10-dpi with prchIL-17 (*n* = 60). At 21-dpi, the frequency of splenic T cells is shown. Non-parametric Wilcoxon’s test (Mann–Whitney) was used to test significance. Data represent mean ± SD (*n* = 5) at each time point. * (*p* < 0.05) and ** (*p* < 0.01) indicates a significant difference. Cont.: control; MDV-inf: MDV-infected.

The results showed distinct effects of prchIL-17 treatment on the frequencies of various T cell subsets in the context of MDV infection at 10-dpi (Figure 5). Upon MDV infection, there was a significant reduction (*p* < 0.01) in the frequency of γδ T-cells and CD3+ CD8α+ T-cells. Further, prchIL-17 treatment at both 4- and 10-dpi did not significantly alter γδ T-cell frequency in MDV-infected groups (Figure 6A). However, treatment with prchIL-17 significantly increased (*p* < 0.01) the frequency of CD3+CD4+ αβ T-cells in MDV-infected groups at 10-dpi but not at 4-dpi (Figure 5B). There is a notable decrease (*p* < 0.01) in the frequency of CD3+ CD4+ αβ T cells in MDV-infected chickens following treatment with prchIL-17 at 4-dpi compared to 10-dpi (Figure 6B). Furthermore, treatment at 4-dpi of MDV-infected groups with prchIL-17 restored the frequency of CD3+CD8α+ αβ T-cells to control levels (Figure 5C). This was contrasted by a significant increase (*p* < 0.05) in the frequency of spleen CD3+ CD8α+ αβ T cells in MDV-infected chickens treated with prchIL-17 at 4-dpi compared to 10-dpi (Figure 6C).

### 3.5. Changes in Gene Expression Profile Following IL-17 Treatment in MDV-Infected Chickens

**Figure 7:**
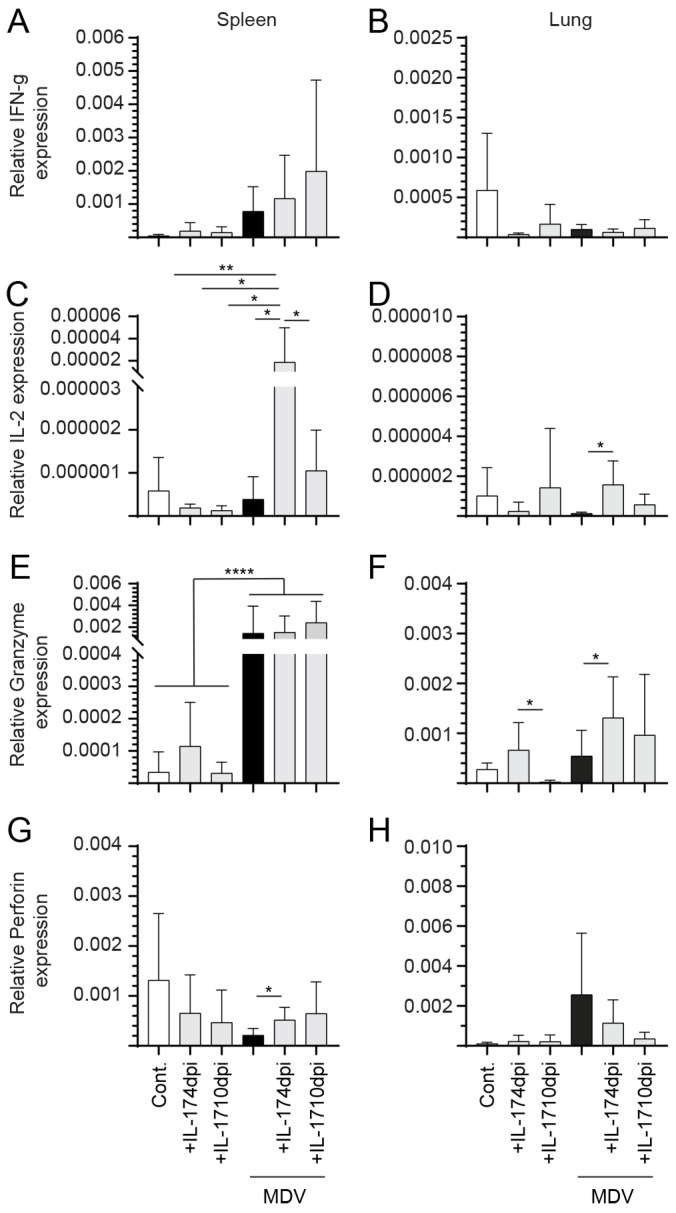
**Expression of cytokines at 21-dpi in vvMDV-RB1B-infected chickens pre-treated at 4- and 10-dpi with prchIL-17.** Gene expression analysis at 21-dpi in vvMDV-RB1B-infected chickens that were pre-treated with the prchIL-17. Expression of the target genes (**A**,**B**) IFN-γ, (**C**,**D**) IL-2, (**E**,**F**) granzyme, and (**G**,**H**) perforin was quantified by real-time PCR and expression is presented relative to the reference gene (β-actin) in the (**A**,**C**,**E**,**G**) spleen and (**B**,**D**,**F**,**H**) lung. The results are based on at least 6 biological replicates in each group. Non-parametric Wilcoxon’s test (Mann–Whitney) was used to test significance, with the results shown as mean ± standard deviation. * (*p* < 0.05), ** (*p* < 0.01), and **** (*p* < 0.001) indicates a significant difference. Cont.: control; MDV-inf: MDV-infected.

The results illustrate the impact of IL-17 treatment on the expression levels of various cytokines (IL-2, IFN-γ, perforin, and granzyme B) in the spleen and lung tissues within MDV-infected chickens at 21-dpi (Figure 7).

Notably, at 21-dpi, expression of IL-2 in the spleen was significantly higher within the MDV-infected group that was treated with prchIL-17 at 4-dpi when compared to the untreated control (*p* < 0.01), control + IL-17 4-/10-dpi (*p* < 0.05), and MDV + IL-17 10-dpi (*p* < 0.05) groups (Figure 7A). Within the lung, IL-2 expression was significantly higher (*p* < 0.05) in the MDV-infected group that was treated with prchIL-17 at 4-dpi compared to the MDV-infected group (Figure 7B). In the spleen (Figure 7C) and lung (Figure 7D), there was no significant difference in IFN-γ expression between all treatment groups at 21-dpi.

Granzyme expression in the spleen of MDV-infected chickens at 21-dpi was markedly increased (*p* < 0.0001) within the groups that were treated at both 4- and 10-dpi with prchIL-17 compared to all uninfected control groups (Figure 7E). No difference in granzyme expression within the MDV-infected group or uninfected control groups was observed (Figure 7E). In the lung, expression of granzyme was significantly higher (*p* < 0.05) in the control groups that were treated with the prchIL-17 at 4-dpi compared to 10-dpi (Figure 7F). Within the MDV-infected groups, expression of granzyme was significantly higher (*p* < 0.05) in the untreated MDV-infected group compared to the MDV-infected group that was treated at 4-dpi with the prchIL-17-expressing plasmid (Figure 7F).

Conversely, perforin expression in the spleen significantly decreased (*p* < 0.05) in the group treated with prchIL-17 at 10-dpi compared to the control (Figure 7G). In the lung, no change was observed in the expression level of perforin across all groups (Figure 7H). In the spleen of all MDV-infected groups at 21-dpi, the expression of perforin was significantly higher (*p* < 0.05) in the group treated with prchIL-17 at 4-dpi but not in chickens treated at 10-dpi or the untreated group (Figure 7G). At 21-dpi, there was no significant difference in the expression of perforin across all treatment groups within the lungs (Figure 7H).

## 4. Discussion

Produced by Th17 cells, the proinflammatory cytokine IL-17 plays an important role in both systemic and mucosal immunity [1,3,4]. While IL-17-producing Th17 cells are well-defined in mice and humans, little is known about the functional role of Th17 cells and IL-17 in avian species [8]. Recently, the expression of the IL-17 gene was demonstrated in mitogen-stimulated avian T cells and further detected via intracellular cytokine staining in various T-cell subsets [25]. Therefore, the aim of this study was to explore the importance of IL-17 for immunity against Marek’s disease in chickens. Our findings provide new insights into the role of IL-17 based on the following results: (i) in vivo treatment with IL-17 at 4-dpi resulted in a reduction in MD lesion severity but not tumor incidence; (ii) in vivo treatment with IL-17 at 4-dpi led to a reduction in CD4+ αβ T-cells and an increase in CD8+ αβ T-cells with the highest proportion peaking at 21-dpi; and (iii) in vivo inoculation with IL-17 at 4-dpi resulted in an increase in MDV viral load in the chicken spleen and lung tissues at 10-dpi and 21-dpi.

IL-17 is recognized as essential for inducing protective mucosal immunity against bacteria and fungi. In contrast, little is known about the role of IL-17 in viral infection followed by tumor formation. In terms of the pathogenic effects of IL-17, its expression can exacerbate inflammatory processes, thereby leading to tumor growth [26]. Recently, we have shown the potential adjuvant effects of chIL-17. In vivo inoculation with a plasmid expressing the recombinant chIL-17 cytokine post-HVT vaccination but prior to infection with MDV-RB1B led to a significant reduction in tumor incidence compared to MDV-infected-only, HVT-vaccinated, and vector-treated control groups [8]. This outcome was associated with a reduction in cyclooxygenase (COX)-2, transforming growth factor beta (TGF-β), and MDV-*Meq* gene expression and an increase in IL-12p40 and IFN-γ expression. Transformation and proliferation of MD CD4+ T-cell lymphomas are associated with viral *Meq* and the induction of host factors such as COX-2 and TGF-β [27,28,29,30]. It is not known whether expression of chIL-17 on its own can either exacerbate or ameliorate MD tumor formation during MD progression. We have previously established that treatment with chIL-17 prior to infection with MDV-RB1B led to lower tumor lesion scores compared to MDV-infected chickens [8]. Therefore, the present study focused on defining a link between IL-17 as administered post-MDV-infection at specific stages of MDV pathogenesis. To that end, we used a pCDNA3.1 plasmid vector for overexpressing IL-17 in vivo. We have previously demonstrated that rchIL-17 is biologically active based on a functional interaction between the rchIL-17 cytokine and the cell surface IL-17R on various immune cell subsets, leading to their subsequent activation both in vitro and in vivo [8]. In fact, pre-treatment of chickens with IL-17 led to a significant increase in transcripts of IL-1β and TGF-β but decreased IL-12p40 and IFN-γ transcripts within the spleen of non-infected chickens [8]. One of the limitations of this study is the inability to perform in vivo monitoring of transfected DNA, gene expression kinetics, and protein expression in chickens. While it is understood that IL-17 can induce a positive feedback loop, we have previously observed no difference in IL-17 gene expression, even up to 24 h post-inoculation with the recombinant plasmid [22]. In this study, the overall effect of IL-17 was assessed based on viral replication and immune modulation that could impact end-point gross tumor lesion scoring. The results of the present study demonstrated that, regardless of the timing of intramuscular inoculation with prchIL-17 at 4- and 10-dpi, no effect on tumor incidence was observed across infected groups. Despite the 100% tumor incidence across all MDV-infected groups, treatment with prchIL-17 at 4-dpi but not at 10-dpi significantly reduced disease severity, as evidenced by the lower lesion scores when compared to MDV-infected groups only or MDV-infected control plasmid-treated chickens. This suggests that early IL-17 intervention can modulate the immune system microenvironment to ameliorate pathological outcomes associated with MDV. The absence of such an effect at 10-dpi highlights a narrow window for IL-17 efficacy. There are at least two possible mechanisms that may be involved in the observed reduction in disease severity: (1) IL-17 may well stimulate immune system cell activation; and (2) IL-17 can directly or indirectly inhibit MDV replication.

Classically, IL-17 facilitates the clearance of multiple bacterial and fungal pathogens, while the role of IL-17 cytokines during viral infections is less understood. Recent studies have evaluated the function of IL-17 during viral infections [31]. In either DNA or RNA viral infection studies, IL-17 can have both a protective and/or a pathogenic involvement [31]. In the mouse model, IL-17A has been shown to promote CD8+ T-cell cytotoxicity against West Nile virus (WNV)-infected cells, thereby resulting in a reduction in viral burden and an increase in host survival rate [19]. In chickens, an in vitro study demonstrated that a rchIL-17 cytokine can induce an antiviral response against avian influenza virus (AIV)-infected chicken fibroblast cells [32]. In the context of herpesviruses, IL-17 has been shown to support viral replication. In fact, in both Epstein–Barr virus (EBV) and Kaposi’s sarcoma-associated herpesvirus (KSHV), IL-17 supports the establishment of chronic viral infection and eventual cancer formation [33]. As such, one of the key factors explored in this study was to define the role of IL-17 in MDV-viral infection. In line with observations from the effect of IL-17 on DNA viruses, our study demonstrated that treatment with prchIL-17 at 4- and 10-dpi resulted in an increase in the MDV-RB1B *Meq* gene copy number in the spleen and lung but not in the skin at 21-dpi. The observed increase in RB1B *Meq* expression, particularly in the spleen at 10- and 21-dpi, indicates that IL-17 may have a role in facilitating MDV replication. We have previously shown that either IL-17 or infection with MDV can induce expression of both COX-2 and TGF-β in vitro and in vivo [8]. Both COX-2 and TGF-β have been implicated in creating an immunosuppressive milieu, thereby supporting both MD tumor formation and MDV-viral replication [15,27,28]. Therefore, in chickens treated with IL-17 at 4-dpi but not 10-dpi, the higher MDV-RB1B viral load in the spleen and lung was not associated with an increase in tumor formation. This suggests that while tumor lesions were less severe, viral replication was not impeded. This indicates an intricate role of IL-17 in modulating viral dynamics.

One of the hallmarks of MDV infection is a semi-productive lytic viral replication that occurs within 2–7-dpi [12,34]. This is followed by a latency phase between 7- and 10-dpi [11]. During the early lytic (between 2–7-dpi) viral replication phase, it is thought that B cells are predominantly affected [35]. Subsequently, MDV-infected B cells are thought to produce the MDV vIL-8 protein that acts as a chemotactic factor to recruit immune cells such as T cells to the site of primary infection and, therefore, facilitate viral dissemination from B cells to T cells [14,36,37,38]. MDV establishes a latent infection in CD4+ T-cells, which defines the latency phase and immune evasion between 7- and 10-dpi [12,34]. The differential outcomes based on observed disease severity between early (4-dpi) and late (10-dpi) administration of the IL-17 cytokine could be related to the specific lytic viral replication cycle and latency phase, respectively. In mammalian models, IL-17 has been associated with increased survival and proliferation of both B and T cells [5,39]. It is known that IL-17 promotes the expression of pro-survival molecules, such as Bcl-2 and Bcl-xL, by utilizing STAT3. The increased expression of these survival molecules plays a critical role in establishing viral persistence and the development of inflammatory diseases and cancers by permitting the survival of virus-infected or pathogenic cells [40,41,42]. Similarly, IL-17 supports the survival of peripheral neurons during herpes simplex virus-2 reactivation [43]. While in the present study we did not assess the proportion of B and T cells that are either dead or apoptotic, it is likely that IL-17 reduced the immunosuppressive effects of lytic viral replication in vivo. Therefore, increasing the longevity of B and T cells can reduce the immunosuppressive effects of lytic viral replication and increase a T-cell-mediated antiviral defense in the chicken. As such, we observed a dynamic shift in specific T-cell subsets following treatment with IL-17. The results presented here demonstrate a significant increase in CD8+ αβ T-cells and a significant decrease in CD4+ αβ T-cells in the spleen of MDV-infected chickens treated with IL-17 at 4-dpi compared to the MDV-infected control group or the control group infected with MDV-infected and treated with plasmid.

The treatment of chickens with prchIL-17 at 10-dpi targeted the latency and immune evasion phases rather than the period of active viral replication [11]. The frequency of CD4+ αβ T-cells in chickens treated with IL-17 at 4-dpi was similar to that in non-infected control chickens. In CD8+ αβ T cells, no difference was observed between non-infected control chickens and chickens treated with IL-17 at 4-dpi. The increased percentage of CD8α+ T cells suggests that IL-17 may play a role in enhancing the cytotoxic potential against MDV. CD8α+ T cells are essential for killing virus-infected cells, and their increase following IL-17 treatment could be pivotal in controlling the spread of the virus [17,44]. In fact, we have previously reported the importance of both IFN-γ+ CD8+ T-cells and IL-17+ T-cells during MDV pathogenesis [7,8,45]. Administration of either a chicken IFN-γ or IL-17 cytokine with an MDV vaccine has been shown to increase vaccine efficacy, suggesting that either IFN-γ or IL-17 may play an important role in boosting protection against MD.

T-cell activation can be defined based on the expression of IL-2, IFN-γ, perforin, and granzyme. Our results further demonstrate an increase in IL-2, perforin, and granzyme but not IFN-γ, supporting T-cell activation. In fact, we have previously observed that treatment with IL-17 led to a decrease in IFN-γ expression [8]. IL-17 and IFN-γ play diverse roles and can modulate differentiation of distinct Th1 and Th17 lineages, respectively [46]. However, the unchanged frequency of γδ T cells indicates a selective effect of IL-17 on different T-cell subsets. This selectivity could be due to distinct signaling pathways and functional roles of γδ versus αβ T cells in the immune response to MDV, which highlights the complex immunomodulatory effects of IL-17. This interplay further supports the findings on disease severity and tumor incidence, indicating that timing is crucial in defining the role of IL-17 as either a modulator or an exacerbator of infection.

In summary, the present study provides new insights into the role of IL-17 in modulating avian immune responses during MDV infection. The differential effects of IL-17 on disease severity, T-cell subset frequencies, and cytokine expression underscore a double-edged sword effect, having both protective and potentially deleterious effects depending on the timing of administration and the specific immune pathways influenced. These findings pave the way for developing targeted immune-based strategies leveraging the modulatory potential of IL-17 in immunopathology and vaccine development.

## Figures and Tables

**Table 1:** Primer sequences for real-time PCR.

Target Gene	Primer Sequence		Annealing Temp	Accession Number
**β-actin**	FWD	5′-CAACACAGTGCTGTCTGGTGGTA-3′	58 °C	X00182
	REV	5′-ATCGTACTCCTGCTTGCTGATCC-3′		
**meq**	FWD	5′-GTCCCCCCTCGATCTTTCTC-3′	64 °C	AY571783
	REV	5′-CGTCTGCTTCCTGCGTCTTC-3′		
**IFN-γ**	FWD	5′-ACACTGACAAGTCAAAGCCGCACA-3′	60 °C	X99774
	REV	5′-AGTCGTTCATCGGGAGCTTGGC-3′		
**IL-2**	FWD	5′-TGCAGTGTTACCTGGGAGAAGTGGT-3′	58 °C	NM_204153.2
	REV	5′-ACTTCCGGTGTGATTTAGACCCGT-3′		
**granzyme A**	FWD	5′-TGGGTGTTAACAGCTGCTCATTGC-3′	55 °C	NM_204457.2
	REV	5′-CACCTGAATCCCCTCGACATGAGT-3′		
**perforin**	FWD	5′-ATGGCGCAGGTGACAGTGA-3′	64 °C	XM_046929135.1
	REV	5′-TGGCCTGCACCGGTAATTC-3′		

## Data Availability

The original contributions presented in this study are included in the article. Further inquiries can be directed to the corresponding author(s).
